# Safety and patients’ satisfaction of transcutaneous Supraorbital NeuroStimulation (tSNS) with the Cefaly® device in headache treatment: a survey of 2,313 headache sufferers in the general population

**DOI:** 10.1186/1129-2377-14-95

**Published:** 2013-12-01

**Authors:** Delphine Magis, Simona Sava, Tullia Sasso d’Elia, Roberta Baschi, Jean Schoenen

**Affiliations:** 1 Headache Research Unit, University Department of Neurology, CHR Citadelle, Boulevard du 12eme de Ligne 1, 4000 Liege, Belgium

**Keywords:** Transcutaneous peripheral nerve neurostimulation, Preventive migraine therapy, Cefaly®

## Abstract

**Background:**

Transcutaneous supraorbital nerve stimulation (tSNS) with the Cefaly® device was recently found superior to sham stimulation for episodic migraine prevention in a randomized trial. Its safety and efficiency in larger cohorts of headache sufferers in the general population remain to be determined.

The objective of this study was to assess the satisfaction with the Cefaly® device in 2,313 headache sufferers who rented the device for a 40-day trial period via Internet.

**Methods:**

Only subjects using specific anti-migraine drugs, and thus most likely suffering from migraine, were included in the survey. Adverse events (AEs) and willingness to continue tSNS were monitored via phone interviews after the trial period. A built-in software allowed monitoring the total duration of use and hence compliance in subjects who returned the device to the manufacturer after the trial period.

**Results:**

After a testing period of 58.2 days on average, 46.6% of the 2,313 renters were not satisfied and returned the device, but the compliance check showed that they used it only for 48.6% of the recommended time. The remaining 54.4% of subjects were satisfied with the tSNS treatment and willing to purchase the device. Ninety-nine subjects out of the 2,313 (4.3%) reported one or more AEs, but none of them was serious. The most frequent AEs were local pain/intolerance to paresthesia (47 subjects, i.e. 2.03%), arousal changes (mostly sleepiness/fatigue, sometimes insomnia, 19 subjects, i.e. 0.82%), headache after the stimulation (12 subjects, i.e. 0.52%). A transient local skin allergy was seen in 2 subjects, i.e. 0.09%.

**Conclusions:**

This survey of 2,313 headache sufferers in the general population confirms that tSNS with is a safe and well-tolerated treatment for migraine headaches that provides satisfaction to a majority of patients who tested it for 40 days. Only 4.3% of subjects reported AEs, all of them were minor and fully reversible.

## Clinical relevance summary

Transcutaneous supraorbital neurostimulation with the Cefaly® device is a safe and satisfactory treatment modality for migraine headache sufferers in the general population who tested it for 40 days. Treatment failure may be partly due to poor compliance.

## Background

Migraine is a highly prevalent primary headache disorder and one of the most disabling diseases worldwide according to the recent epidemiologic data [1]. Preventive anti-migraine drug therapies have incomplete efficacy and many of them have cumbersome side effects [2]. Blumenfeld et al. (2013) [3] have recently found that only 28.3% and 44.8% of subjects suffering respectively from the episodic and chronic forms of migraine (ICHD-III beta criteria 1.1, 1.2 and 1.3 [4]) were currently using a preventive medication [3]. The reasons for treatment discontinuation were lack of efficacy and side effects in an equal proportion. Furthermore, over the last decade hardly any novel migraine preventive drug has been marketed. Hence, there is a need for new preventive therapies with similar or better clinical efficacy, and most importantly fewer treatment-related side effects.

Peripheral nerve stimulation (PNS) has shown promising preventive properties in episodic and chronic migraine [5]. PNS conveys its effects by the electrical stimulation of peripheral nerves branches either sub- or percutaneously with implantable devices, or transcutaneously via superficial skin electrodes linked to external neurostimulators. Due to its invasiveness percutaneous PNS like occipital nerve stimulation (ONS) was used hitherto only in the most disabled migraine patients [5-7]. Transcutaneous PNS have the advantage of being non-invasive and thus applicable also in less severely disabled subjects suffering from episodic migraine.

We have shown previously in a randomized double-blind sham-controlled trial that transcutaneous supraorbital neurostimulation (tSNS) is effective in the preventive treatment of episodic migraine (the PREMICE trial, [8]). In this study, subjects were treated with an external ultra-portable and user-friendly tSNS device stimulating both supraorbital nerves, the Cefaly® device (CEFALY Technology, Herstal, Belgium). After daily 20 minutes tSNS sessions for 3 months, the 50% responder rate was 38.2% for active tSNS vs.12.1% for sham stimulation [8]. The effect was significant, and within the range of other migraine preventive therapies. Moreover, there were no side effects or drop-outs due to device-related adverse events.

However, the number of subjects included in this trial was limited to 67 patients recruited in tertiary headache clinics. It remains therefore to be studied how tSNS with the Cefaly® device performs in larger cohorts of headache sufferers in the general population. For this purpose, we have conducted a survey of subjects who rented the device via Internet for 40 days, in order to assess safety and satisfaction of tSNS in a large cohort of more than 2000 headache sufferers.

## Methods

### Subjects

A prospective registry of 2,573 headache sufferers who rented the tSNS Cefaly® device (CEFALY-Technology, Liège, Belgium) was established between September 2009 and June 2012. Most subjects were French or Belgian citizens, while a minority lived in Switzerland, three countries where subjects can directly rent and buy the device via the Internet without medical prescription. The device can be rented at a cost of 49€ for 40 days, where after the patient has to decide either to return the device or to keep it and pay the balance between its cost of 295€ and the rental fee.

### Transcutaneous supraorbital neurostimulation

**Figure 1:**
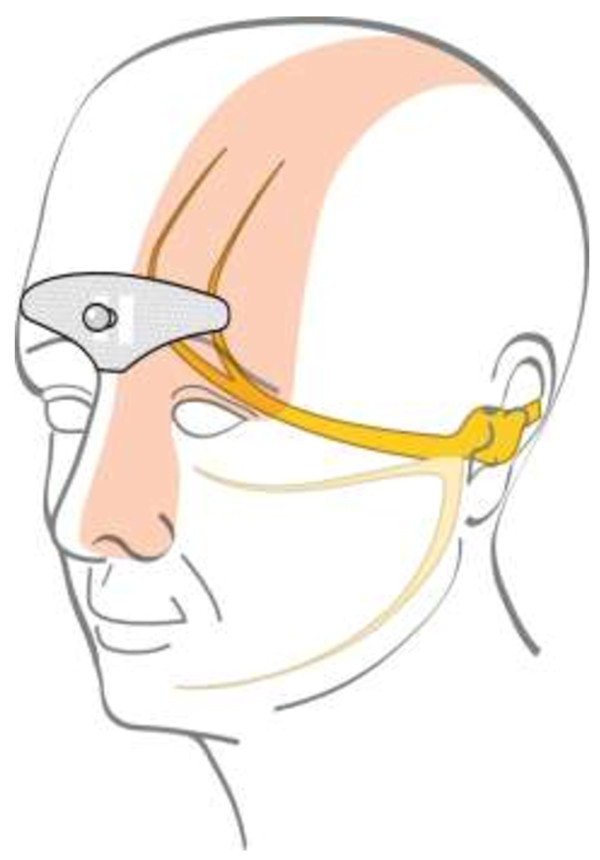
Area covered by the tSNS electrode.

tSNS was delivered with an external self adhesive electrode placed on the forehead (Figure 1, Cefaly® device, CEFALY Technology, Liège, Belgium). The bipolar electrode (30 mm × 94 mm) covers bilaterally the origins of the supraorbital nerves (branches of 1st trigeminal division). A constant current generator (maximum skin impedance of 2.2 KΩ) generates biphasic rectangular impulses with an electrical mean equal to zero (preventive stimulation protocol: impulse width 250 μS, frequency 60 Hz, maximum intensity 16 mA). All subjects received an explicative leaflet advising to perform tSNS at least once daily in order to obtain a preventive anti-migraine effect. As single sessions have a fixed duration of 20 minutes, the recommended minimal total time of use was 800 minutes in subjects renting the device for 40 days. A built-in electronic system allowed recording of the total time of tSNS use in subjects who returned then device to the manufacturer after the trial period.

### Data collection and processing

The objectives of this survey were to record self-reported adverse events and to assess the satisfaction of subjects who received the tSNS Cefaly® device at home with its accessories for the rental period.

After the end of this rental period they were contacted to answer to the following questions:

1. Which kind of medication do you usually take to treat your headache attack?

2. Did you have side effects when using tSNS or any complaint or comment about the device?

3. Did you encounter technical issues with the device?

4. Are you satisfied with tSNS and do you want continue the treatment?

“Satisfied” subjects who wanted to keep on the treatment had to purchase the device (i.e. to pay 246€), whereas “unsatisfied” subjects sent it back by surface mail.

The devices collected from unsatisfied subjects were analyzed for the total time of tSNS use in order to estimate compliance.

A trained medical secretary was paid by the manufacturer to contact all subjects by phone or e-mail after the rental period. Phone contact was tried in the morning, at noon and in the afternoon; an e-mail was sent in case the person did not answer the phone. This was repeated for up to 2 weeks until a formal contact was achieved.

A total of 2,573 patients rented the device during the 29 months of the survey; 26 never responded to the phone calls or e-mails; 234 were not using triptans and were not included in the survey, as they were assumed not to suffer from migraine. In the three involved countries (Belgium, France and Switzerland) triptans are indeed only delivered and/or reimbursed with a medical prescription certifying that the patient has a diagnosis of migraine according to ICHD-II criteria [9].

**Figure 2:**
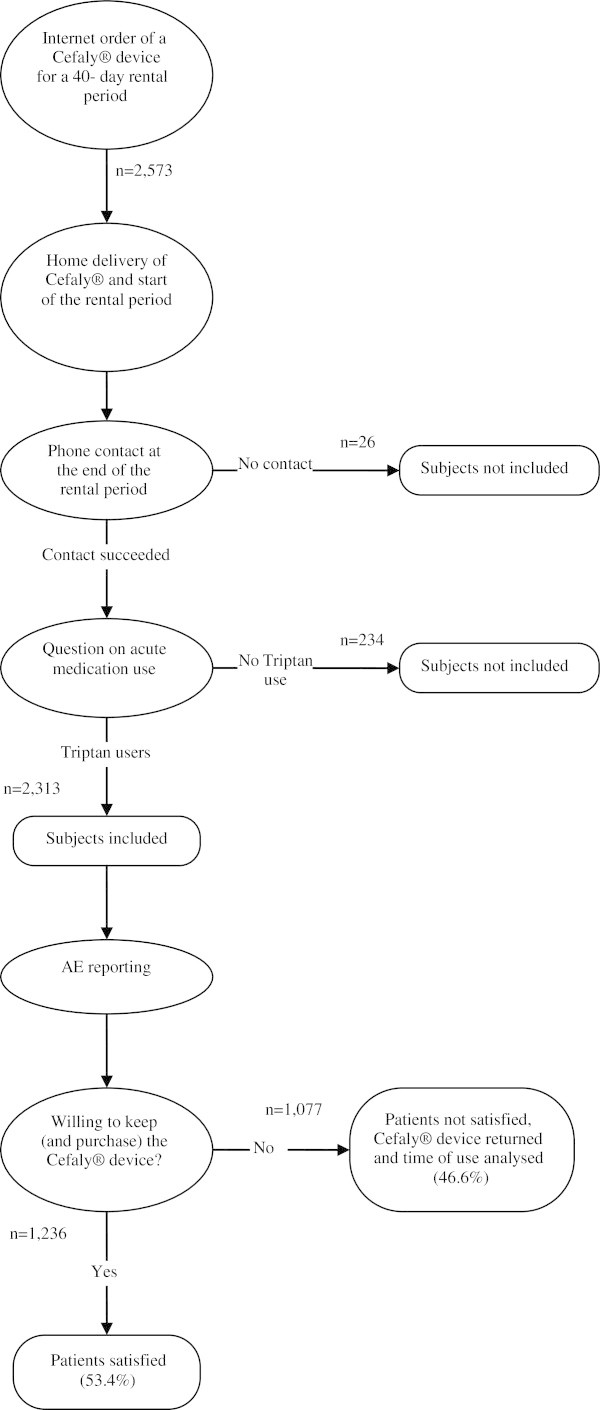
Study flow chart.

The diagram in Figure 2 depicts the sequential steps of the survey.

According to European regulations on non-interventional studies with medical devices (CE directive 93/42 and ISO 13485) this survey did not require ethics committee approval.

## Results

According to the triptan use selection criterion, 2,313 headache sufferers were included in the survey (age 14–87 years, 1641 females i.e. 70.95% and 672 males i.e. 29.05%): 1,208 (52.2%) from France, 999 (43.2%) from Belgium and 106 (4.6%) from Switzerland. The average rental period, computed from the day they received the device until they were actually contacted to answer the questions, was 58.2 ± 33.6 days.

### Safety

Ninety-nine subjects reported at least one adverse event (AE) during tSNS therapy, i.e. 4.3% of all subjects. In the subgroup of unsatisfied patients the AEs rate was 5.48% (59 patients) and it was 3.24% (40 patients) in the subgroup of satisfied patients. Five patients reported more than one AE, one in the satisfied subgroup and four in the unsatisfied subgroup. Forty-six subjects, i.e. 2%, stopped tSNS because of an AE. None was serious and all were fully reversible. The most remarkable AE was a forehead skin allergy in 2 subjects (0.09%).

Table 1 is an exhaustive list of all AEs recorded.

**Table 1:** AE reported by the patients within the trial period

	**Number of patients**	**Percentage of AE**	**Percentage of patients**
Do not like the feeling and do not want to continue using the device	29	29.29%	1.25%
Sleepiness during the Cefaly® session	12	12.12%	0.52%
Headache after a Cefaly® session	12	12.12%	0.52%
Reversible forehead skin irritation	5	5.05%	0.22%
Insomnia	4	4.04%	0.17%
Feeling of fatigue	3	3.03%	0.13%
Persistent forehead paresthesia for several minutes after the session	3	3.03%	0.13%
Feeling of stress during the session	3	3.03%	0.09%
Allergic skin reaction	2	2.02%	0.09%
Dental pain during the session or at the beginning	2	2.02%	0.09%
Inability to keep eyes open during sessions	2	2.02%	0.09%
Feeling of contusion on the forehead during a few days	2	2.02%	0.09%
Pre-existing tinnitus increased during the session	1	1.01%	0.04%
Tinnitus appearing during some sessions	1	1.01%	0.04%
Red eye after a session	1	1.01%	0.04%
Eyes weeping during a session	1	1.01%	0.04%
Wake up during night with a feeling of anxiety and tremor	1	1.01%	0.04%
Vertigo during the first session	1	1.01%	0.04%
Vomiting after a session	1	1.01%	0.04%
Forehead skin burning sensation during a session	1	1.01%	0.04%
Cervical pain during sessions	1	1.01%	0.04%
Cervical pain with nausea after the two first sessions	1	1.01%	0.04%
Short feeling of electrical shock	1	1.01%	0.04%
Slight pain at one eyebrow during the first session	1	1.01%	0.04%
Nausea and vertigo during sessions	1	1.01%	0.04%
Nausea during sessions	1	1.01%	0.04%
More head pain when using the device during a headache	1	1.01%	0.04%
Forehead and cranial anaesthesia feeling during a few hours after a session	1	1.01%	0.04%
Pressure feeling between the eyebrows during sessions	1	1.01%	0.04%
Numbness at the back of the head after a session	1	1.01%	0.04%
Stronger paresthesia feeling on the left side	1	1.01%	0.04%
Stronger paresthesia feeling on the right side	1	1.01%	0.04%
Subjective tachycardia during a session	1	1.01%	0.04%
Migraine feeling during sessions	1	1.01%	0.04%

Complaints reported by patients.

The most frequent AE was intolerance to the paresthesia induced by the electrical stimulation (N = 31, 46% of all AEs), despite the fact that the subjects were allowed to interrupt the gradual intensity increase from 0 to 16 mA by pressing the “on” button as soon as the forehead sensation became uncomfortable. All subjects complaining of paresthesia intolerance stopped the treatment. Some other painful feelings were reported: 3 strong pressure feelings on the forehead, 2 dental and 2 cervical pains during the session. Two subjects felt paresthesia more on one side of the forehead. While the paresthesia stopped in most subjects at the end of the stimulation, 4 individuals reported that the forehead paresthesia persisted for several hours after the end of the stimulation.

Twelve subjects (0.52%) complained after the tSNS session of tension-type like headache that led to treatment interruption.

Arousal and sleep changes were the second most frequently reported AEs (19 subjects or 18.6% of all AEs). Among them, sleepiness during the stimulation was reported by 12 subjects, while 4 complained of insomnia. Three subjects (3.03%) complained of a feeling of stress during the tSNS session.

Three subjects had nausea and vomiting at the end of a session, but did not complain of headache.

Two subjects reported not being able to keep their eyes open during the stimulation. Two reported increased tinnitus during the session, one a red eye and another one tearing.

**Figure 3:**
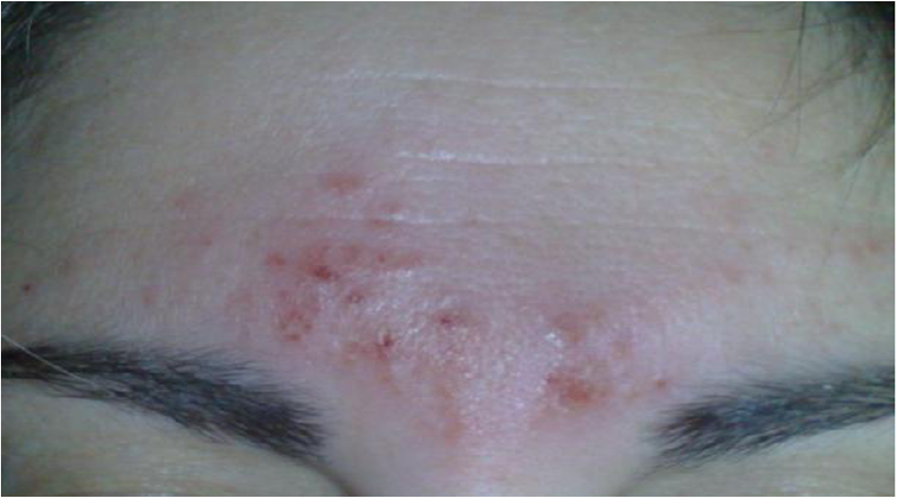
Allergic skin reaction.

Five subjects (5%) complained about transient skin irritation and 2 subjects had a local cutaneous reaction, probably allergic to the electrode gel containing acrylate (2% of all AEs, and 0.09% of all subjects) (Figure 3). These patients did not report a history of allergy to adhesive tapes but one of them had previously suffered from an allergic skin reaction.

The four remaining AEs were single and mild: numbness at the back of the head, slight pain over one eyebrow, feeling of abrupt electrical variation, tachycardia during one session.

### Satisfaction

Out of 2,313 subjects, 1,236 (53.4%) were satisfied with the tSNS therapy and wanted to continue the treatment. These subjects purchased the Cefaly® device. On the opposite, 1,077 subjects (46.6%) were not satisfied with tSNS and sent back the device.

### Compliance

The devices collected back from the 1,077 non-satisfied subjects who discontinued the therapy were analyzed for time of use (Table 2). A built-in electronic system in each device recorded the total time of use. In these 1,077 unsatisfied subjects the mean time of use was 583 ± 903 minutes, for an average rental period of 49.5 ± 26.7 days. As the recommended treatment schedule was one session of 20 minutes per day, their time of use was 58.8% of the recommended time.

**Table 2:** Compliance in the 1,077 unsatisfied subjects

**Total time of use (minutes)**	Number of subjects (percentage)*
0	48 (4,46%)
1 to 20	58 (5,39%)
21 to 40	46 (4,27%)
41 to 60	53 (4,92%)
60 to 100	78 (7,24%)
100 to 200	174 (16,16%)
200 to 400	189 (17,55%)
> 400	431 (40.02%)

*Number (and percentage) of unsatisfied subjects who used the device for the time indicated in the first column i.e. 48 “unsatisfied” subjects did not switch on the device, 58 used the device between 1 and 20 minutes, 46 between 21 and 40 minutes, etc.

Interestingly, 4.46% of “unsatisfied” subjects (N = 48, 2.08% of all subjects) did not even switch on the device, and 19.03% used it less than 60 minutes. Conversely, 40% of the discontinuers applied the tSNS for more than 400 minutes over the rental period (N = 431, 18.63% of all subjects), which was probably sufficient to achieve a therapeutic effect. If we exclude from the survey subjects who never switched on the device, i.e. who did not try the treatment at all, the percentage of satisfied subjects raises to 55.51%. Also, if one accepts that 400 minutes of treatment are necessary to obtain a treatment effect, only 18.63% of all subjects would be classified as non-responders; the compliance of the other discontinuers (N = 646) was not large enough to assess a treatment response.

Out of the 646 patients who used their device less than 400 minutes 56 reported AEs (8.64%), i.e. twice more the AE rate for all subjects. In patients who used the device at least 400 minutes the AE rate was 1.85%.

## Discussion

This survey on a large cohort of 2,313 headache sufferers in the general population provides important data on tolerance and safety of tSNS with the Cefaly® device as well as some information about its performance.

First, it underscores the safety of tSNS and the low incidence of self-reported AEs (4.3% of 2,313 subjects). About half (2%) of these subjects discontinued the therapy because of an AE. In the PREMICE trial [8] the 34 subjects randomized to the effective tSNS (verum) arm reported no AE and none dropped out, which can be explained by the small number of patients. In the present survey the most frequent adverse effect was intolerance to forehead paresthesia that was perceived as painful burning sensations. As a matter of fact, paresthesia is a “normal” sensation linked to every PNS, and responsible for the difficulty in effectively blinding such studies. It is common experience, however, that a number of subjects in the general population do not tolerate the sensations induced by cutaneous electrical stimuli even at low intensities. This intolerance may be pronounced in migraine sufferers and might be related to the cutaneous allodynia that may persist in some of them between attacks [5]. Subjects reporting sleepiness confirm that tSNS can have sedative properties, as shown previously in a study of healthy volunteers [10]. Finally, the most remarkable and cumbersome AE was skin allergy under the forehead electrode (0.09%). Though very rare, such an allergic reaction is well known for self-adhesive electrodes and attributed to the acrylate component of the electrode gel [11]. It is fully reversible within 10 days after removing the electrode and can be avoided by using a newly developed hypoallergenic gel without acrylate.

Second, this survey indicates that 53.4% of subjects were satisfied with the tSNS after a trial period of on average 58 days, and decided to continue the treatment and to purchase the device. Although this is a purely subjective global assessment, patients’ satisfaction could somehow parallel treatment effectiveness. In the PREMICE trial, 70.6% of episodic migraineurs were satisfied with the treatment (29.4% very, 41.2% moderately satisfied) [8]. The global rate of satisfaction is lower in the present survey, but one has to take into account that subjects had to pay 246€, i.e. the difference between the full price and the rental cost, to purchase the Cefaly® to keep the device for treatment continuation, and that the subject population is far more heterogeneous. The compliance to tSNS therapy was 58.8% in subjects who discontinued the treatment while in the PREMICE study it was 61.7% in the total group of patients. This slight difference can be due to the fact that only the devices of unsatisfied subjects could be analyzed for time of use. Moreover, patients included in the PREMICE trial were recruited by established headache specialists and thus well educated in headache management including the use of headache diaries while the majority of subjects included in the present survey had no regular neurological follow-up.

If we exclude from the analysis those 48 unsatisfied subjects who never used the device, the rate of satisfied subjects raises to 55.5%. It is likely that non-satisfied subjects who used the device for less than 400 minutes (N = 646, 27.9% of the 2,313 subjects and 59.98% of the unsatisfied subjects) were not sufficiently dosed to expect a therapeutic effect, although they may have experienced adverse effects. Conversely, those 40% of unsatisfied subjects (18.6% of the 2,313 subjects) who applied tSNS for more than 400 minutes, i.e. for a potentially effective duration, are most probably genuine non-responders.

The survey presented here has several weaknesses. The major one is that we have no certainty about the precise diagnosis of included subjects, which is the reason why we have focused our analysis on safety and tolerance. We assume that a majority of them probably suffered from migraine because they were using triptans for the treatment of headache attacks. In the three involved countries (Belgium, France and Switzerland) triptans are not available over-the counter, but delivered and reimbursed only with a medical prescription certifying that the patient suffers from migraine according to ICHD-II criteria [9]. Triptan users are thus most likely to have been diagnosed as migraineurs by a general practitioner and/or a neurologist. Whether they suffer from episodic or chronic migraine, from migraine with or without aura cannot be determined in our survey. Possible diagnostic confounders are misdiagnosed headache, tension-type headache, medication overuse headache and cluster headache.

Other weaknesses are the absent control for concurrent drug treatment and for natural history of the headache disorder, as well as the outcome parameter and the time point at which it was assessed. As mentioned above, patients’ satisfaction, the only parameter available here, is a composite subjective outcome measure combining efficacy, tolerance, adverse effects, expectations and, in this case, willingness to pay. It is not a recommended primary measure of efficacy, like the number of headache days, and it does not necessarily parallel a reduction in headache frequency. Despite its shortcomings, however, patients’ satisfaction is considered to be valuable in pragmatic trials such as ours, according to the IHS guidelines for controlled trials of drugs in migraine [12]. The time point of about 60 days of tSNS at which the subjects’ satisfaction was assessed may not be optimal. In the PREMICE trial the treatment period was 3 months and the reduction in migraine day frequency was maximal at the end of the 3rd month [8]. The tSNS efficiency may thus be underestimated in our survey, though this would probably concern only a minority of subjects, since the therapeutic advantage over sham stimulation was already significant at the end of the 2nd month of treatment in the PREMICE trial [8]. Finally, we cannot exclude the possibility that some individuals in whom the device was effective did not purchase it for financial reasons, which would have led to an overestimation of the proportion of non-satisfied subjects.

Because of these shortcomings no definitive conclusion about therapeutic efficacy can be drawn from this survey.

## Conclusions

This survey of 2,313 headache subjects treated with tSNS is to the best of our knowledge the largest database available for a neuromodulation treatment in headache. Its major contribution is to confirm the safety and excellent tolerance of tSNS therapy with the Cefaly® device. Adverse events were reported by only 4.3% of subjects and they were all minor and reversible. The most frequent AE was intolerance to the local paresthesia, which is a common, though rare, reason for treatment interruption in every PNS therapy. About 2% of subjects stopped the tSNS therapy because of an AE, which is remarkably low compared to preventive anti-migraine drugs [3]. Although this survey does not allow reliable deductions about efficacy for methodological reasons, it provides some clinically useful indications about patients’ satisfaction and compliance. Among the 2,313 subjects, 53.4% were satisfied with the treatment and the device, and decided to buy it. The mean time of tSNS use in those subjects who discontinued the therapy was 58.8% of the recommended time; 4.46% of “unsatisfied” subjects did not even switch on the device, and 19.03% used it for less than 60 minutes. Hence, low compliance to tSNS is an issue that might explain lack of efficacy in a number of subjects.

## Abbreviations

AE: Adverse event; ICHD-II: International classification of headache disorders – 2nd edition; PNS: Peripheral nerve stimulation; PREMICE: PREvention of MIgraine by supraorbital transcutaneous neurostimulation using the CEfaly device; tSNS: Transcutaneous supraorbital neurostimulation.

## Competing interests

The authors declare that they have no competing interests.

## Authors’ contributions

SS, TSD and RB participated in data collection, management and analysis. DM analysed the final data. DM and JS wrote the manuscript. All authors read and approved the final manuscript.
